# Risk classification by pathological and biochemical prognostic factors determined by extensive exploration for metastatic hormone sensitive prostate cancer

**DOI:** 10.1007/s00345-025-05862-4

**Published:** 2025-08-11

**Authors:** Keisuke Goto, Kohei Kobatake, Kenichiro Fukuoka, Yoshito Kagiyama, Tomoya Hatayama, Fumiaki Kirishima, Kazuma Yukihiro, Yoshimasa Kurimura, Takumi Ikai, Kohei Saito, Satoshi Shirane, Hiroaki Yasumoto, Nobuyuki Hinata

**Affiliations:** 1 Department of Urology, Hiroshima University Graduate School of Biomedical and Health Sciences, 1-2-3 Kasumi, Minami-ku, Hiroshima, 734-8551 Japan; 2 Department of Urology, NHO Kure Medical Center and Chugoku Cancer Center, Kure, Japan; 3 Department of Urology, NHO Higashihiroshima Medical Center, Higashihiroshima, Japan; 4 Department of Urology, Matsuyama Red Cross Hospital, Matsuyama, Japan; 5 Department of Urology, Hiroshima City North Medical Center Asa Citizens Hospital, Hiroshima, Japan; 6 Department of Urology, NHO Fukuyama Medical Center, Fukuyama, Japan; 7 Department of Urology, Hiroshima Prefectural Hospital, Hiroshima, Japan; 8 Department of Urology, Chugoku Rosai Hospital, Kure, Japan; 9 Department of Urology, Miyoshi Central Hospital, Miyoshi, Japan; 10 Department of Urology, JA Onomichi General Hospital, Onomichi, Japan; 11 Department of Urology, NHO Hiroshima-Nishi Medical Center, Otake, Japan

**Keywords:** Prostate cancer, mHSPC, Real-world data, Cancer, Epidemiology

## Abstract

**Purpose:**

To determine prognostic parameters, we extensively examined whether physical, biochemical, and histological factors were associated with clinical outcomes in metastatic hormone sensitive prostate cancer (mHSPC) patients.

**Methods:**

A total 822 mHSPC patients were retrospectively investigated and examined the associations between prognosis and clinicopathological parameters including BMI, initial PSA level, TNM classification, Hb, Alb, CRP, AST, ALT, LDH, ALP, Gleason grade group, and EOD score.

**Results:**

According to the CHAARTED criteria, 338 (41.1%) and 484 (58.9%) patients were classified into low- and high-volume disease, respectively. In univariate and multivariate analyses, Gleason grade group, Alb, CRP, LDH, and ALP were determined as significant predictors for both PFS and OS. When mHSPC patients were classified into three group including favorable (none of risk factors), intermediate (one or two risk factors) and poor (more than three risk factors) according to these four parameters, the survival curves were significantly stratified according to the risk classification. When the risk classification was applied on the patients with low- or high-volume disease in CHAARTED criteria, worse prognosis was found in poor risk group patients with low-volume disease and favorable prognosis was found in favorable risk group patients with high-volume disease.

**Conclusion:**

These results suggested that Gleason grade, CRP, LDH, and ALP were the independent predictors for mHSPC patients regardless of metastatic burden.

**Supplementary Information:**

The online version contains supplementary material available at 10.1007/s00345-025-05862-4.

## Introduction

Prostate cancer (PCa) is the most common male malignant neoplasm worldwide [1]. Thus far, most of the patients with PCa have been determined as organ confined disease and have opportunities to receive curative treatment because of the development of diagnostic modalities. However, metastatic PCa has still been found in approximately 10–15% of the patients and systemic chemotherapy and combination regimen using androgen receptor signal inhibitor (ARSI) would be indicated [2, 3]. Initially, metastatic PCa was regarded as metastatic hormone sensitive PCa (mHSPC) because of its androgen dependency. Although mHSPC had good response to androgen deprivation therapy (ADT), most of the cases would acquire hormone resistance and become castration resistant PCa (CRPC) [4]. Because the prognosis of CRPC has still been unsatisfied, several lines of clinical trials conducted to suggest more effective primary treatments for mHSPC to delay subsequent hormone refractory disease [5, 6]. The CHAARTED trial, regarding upfront docetaxel plus ADT, demonstrated effectiveness of the treatment according to low or high tumor volume [7]. Besides, the LATITUDE trial, regarding upfront abiraterone acetate plus ADT, was performed in mHSPC patients with high risk factors including histological (Gleason score ≥ 8) and metastatic status (more than three bone metastases or visceral metastasis) [8, 9]. It has also been reported that lactate dehydrogenase (LDH), alkaline phosphatase (ALP), and aspartate transaminase (AST)/alanine transaminase (ALT) ratio were associated with the prognosis of advanced PCa with bone metastasis [10]. These clinical studies suggested that the certain factors might be associated with the clinical outcomes of mHSPC. In this study, we extensively examined whether physical, biochemical, and histological factors were associated with clinical outcomes in real-world datasets of mHSPC patients.

## Materials and methods

### Patients and clinical data collection

This retrospective study was conducted as a part of Hiroshima Cancer Registry project (H-CARP) including Hiroshima University hospital and affiliated institutions. A total of 886 mHSPC cases were retrospectively reviewed. Because 64 cases were excluded due to lack of data or follow-up information, 822 cases were included in this study. The present study received the approval from the ethics committee of Hiroshima University (authorization number: E2021-2519). The study data of patients were managed using Research Electronic Data Capture (REDCap) electronic data capturing system hosted at Hiroshima University [11, 12]. The clinical information was collected retrospectively from the medical records of this cohort. The clinical parameters included age, body mass index (BMI), initial PSA level at diagnosis, TNM classification and baseline data of peripheral blood tests. The peripheral blood data included neutrophil counts, hemoglobin (Hb), albumin (Alb), C-reactive protein (CRP), aspartate transaminase (AST), alanine transaminase (ALT), lactate dehydrogenase (LDH) and alkaline phosphatase (ALP) at initial diagnosis of mHSPC. All patients underwent prostate needle biopsy and were assessed according to the International Society of Urological Pathology (ISUP) 2014 modified Gleason grading group [13].

### Staging and assessment of metastatic burden

All patients in this study underwent prostate biopsy for the diagnosis and determined as acinar adenocarcinoma of prostate by histological examination. The needle for prostate biopsy was used 18 gauge (1.2 mm) in diameter. As imaging examinations, computed tomography and bone scintigraphy were performed to investigate lymph node metastasis, distant metastasis, and bone metastasis. TNM classification was used to decide the clinical stage. The metastatic burden was evaluated according to CHAARTED criteria. The high-volume disease was defined if the presence of visceral metastases or ≥ 4 bone lesions with ≥ 1 beyond the vertebral bodies and pelvis was evident. The number of bone metastasis was counted by bone scintigraphy. The EOD score, that was suggested as convenient and semi-quantitative assessment tool of bone metastases, was defined as follows: 1; <6 hot spots, 2; 6–20 hot spots, 3; >20 hot spots but not super scan, 4; super scan, according to the previous reports [14, 15].

### Statistical analyses

Clinical outcomes were evaluated based on progression free survival (PFS) and overall survival (OS) using Kaplan-Meier methods and p-value were calculated by log-rank test. The disease progression was defined according to Prostate Cancer Working Group 3 criteria, when either PSA elevation or radiographic progression was evident. The univariate and multivariate analyses were performed using cox proportional hazard model to investigate the significant predictors for PFS and OS. Also, the stepwise method was used to determine the suitable factors for the risk classification. All statistical analyses were performed using JMP pro 17.0 (SAS Institute Inc., Cary, NC) with significant set at *p* < 0.05.

## Results

### Patient characteristics

The background and characteristics of 822 mHSPC patients are summarized in Table 1. The median age, BMI and PSA level at diagnosis were 73 years old, 22.8 kg/m^2^ and 221 ng/mL, respectively. Histologically, 507 (61.7%) out of 822 patients were diagnosed with Gleason grade group 5 (GG5). In TNM classification, there were 226 (27.5%) of T4 and 488 (59.4%) of N1, 71 (8.6%) of M1a, 613 (74.6%) of M1b and 138 (16.8%) of M1c patients, respectively. In 751 patients with M1b or M1c, 290 (38.6%) patients had only bone metastasis, 323 (43.0%) patients had lymph node and bone metastases, 16 (2.1%) patients had only visceral metastasis, 20 (2.7%) patients had lymph node and visceral metastases, 25 (3.3%) patients had bone and visceral metastases, and 77 (10.3%) patients had lymph node, bone, and visceral metastases, respectively. When the bone metastases were evaluated by EOD score, 178 (21.7%) patients were categorized as EOD 3 or 4. The median values of neutrophil count, Hb, Alb, CRP, AST, ALT, LDH and ALP were 4131 /µL, 13.5 g/dL, 4.1 g/dL, 0.2 mg/dL, 24 U/L, 17 U/L, 203 U/L and 341 U/L, respectively. According to the CHAARTED criteria, 338 (41.1%) and 484 (58.9%) patients were classified into low- and high-volume disease, respectively. As the primary treatment, 690 (83.9%) patients were treated with ADT plus bicalutamide, and the others were treated with upfront ARSI regimen. The median follow-up period was 36 months.

**Table 1:** Patients’ characteristics

No. of patients	Total (N=822)	ADT/CAB (N=692)	Upfront ARSI (N=130)	p-value
Age (median, range)	73 (46–93)	74 (48–96)	72 (47–90)	0.281
BMI (median, range), kg/m^2^	22.8 (14–34.9)	22.8 (14–34.9)	22.9 (16.8–30.8)	0.214
Initial PSA (median, range), ng/mL	221 (4.64–101000)	200 (4.64–101000)	341 (4.8–41041)	0.182
Gleason grade, n (%)				
Group 1–4	315 (38.3)	275 (39.7)	40 (30.8)	0.086
Group 5	507 (61.7)	417 (60.3)	90 (69.2)	
Clinical T stage, n (%)				
T1–T3	596 (72.5)	511 (73.8)	85 (65.4)	0.052
T4	226 (27.5)	181 (26.2)	45 (34.6)	
Clinical N stage, n (%)				
N0	334 (40.6)	291 (42.1)	43 (33.1)	0.053
N1	488 (59.4)	401 (57.9)	87 (66.9)	
Clinical M stage, n (%)				
M1a	71 (8.6)	62 (9.0)	9 (6.9)	0.261
M1b	613 (74.6)	520 (75.1)	93 (71.5)	
M1c	138 (16.8)	110 (15.9)	28 (21.5)	
EOD score, n (%)				
0–2	644 (78.3)	562 (81.2)	89 (68.5)	0.002
3-4	178 (21.7)	130 (18.8)	41 (31.5)	
CHAATED criteria				
Low volume	338 (41.1)	307 (44.4)	31 (23.8)	< 0.001
High volume	484 (58.9)	385 (55.6)	99 (76.2)	
Peripheral blood test (median, range)				
Neutrophil, /µL	4131 (811–36263)	4004 (1231–36323)	4236 (811–13452)	0.483
Hb, g/dL	13.5 (4.0–19.0)	13.5 (4.0–19.0)	13.6 (8.6–17.7)	0.531
Alb, g/dL	4.1 (0.8–5.1)	4.05 (0.8–5.1)	4.1 (2.3–4.7)	0.645
CRP, mg/dL	0.20 (0–26.6)	0.20 (0–26.6)	0.14 (0–22.3)	0.151
AST, U/L	24 (2–203)	24 (2–170)	23 (6–203)	0.295
ALT, U/L	17 (5–159)	17 (5–159)	17 (6–82)	0.789
LDH, U/L	203 (114–4673)	204 (114–4673)	198 (138–534)	0.905
ALP, U/L	340 (65–30318)	331 (65–30318)	372 (80–4611)	0.450
Second treatment, n (%)				
Bicalutamide to ARSI	286	286	0	
Bicalutamide to Docetaxel	68	68	0	
ARSI to ARSI	17	0	17	
ARSI to Docetaxel	15	0	15	

### Survival analyses for PFS and OS

To investigate clinicopathological parameters that were associated with the prognosis of mHSPC, PFS and OS were evaluated by Kaplan-Meier analysis. There were significant associations between BMI (*p* < 0.001), PSA (*p* < 0.001), Gleason grade group (GG)5 (*p* < 0.001), T stage (*p* < 0.001), N stage (*p* < 0.001), EOD score (*p* < 0.001), Neutrophil (*p* = 0.003), Hb (*p* < 0.001), Alb (*p* < 0.001), CRP (*p* < 0.001), AST/ALT (*p* < 0.001), LDH (*p* < 0.001), and ALP (*p* < 0.001) and PFS (Supplementary Fig. S1). Regarding OS, there were significant associations in age (*p* = 0.017), BMI (*p* < 0.001), GG5 (*p* < 0.001), EOD score (*p* < 0.001), Hb (*p* < 0.001), Alb (*p* < 0.001), CRP (*p* < 0.001), AST/ALT ratio (*p* < 0.001), LDH (*p* < 0.001) and ALP (*p* < 0.001) in log rank test (Supplementary Fig. S2). These results confirmed that such clinicopathological parameters were associated with the prognosis of mHSPC.

### Development and validation of a model for predicting the risk of mHSPC

Because a big model with too many clinicopathological parameters might unfit to external datasets, we tried to narrow down those factors to develop a feasible model for predicting the risk of mHSPC. A stepwise regression model revealed that GG5, CRP, LDH, and ALP were determined as the most suitable factors for OS. To evaluate whether those factors were confounded with the other parameters including visceral metastasis, CHAARTED classification, and treatment regimen, Cox proportional hazards model was used. Univariate analysis showed that GG5 (HR = 1.77, *p* < 0.001), CRP (HR = 1.98, *p* < 0.001), LDH (HR = 2.34, *p* < 0.001), ALP (HR = 2.11, *p* < 0.001), CHAARTED classification (HR = 1.81, *p* < 0.001), and upfront ARSI regimen (HR = 0.56, *p* < 0.001) were significantly associated with PFS. Multivariate analysis showed that GG5 (HR = 1.69, *p* < 0.001), CRP (HR = 1.61, *p* < 0.001), LDH (HR = 1.43, *p* = 0.007), ALP (HR = 1.56, *p* < 0.001), CHAARTED classification (HR = 1.52, *p* < 0.001), and upfront ARSI regimen (HR = 0.44, *p* < 0.001) were significantly associated with PFS (Table 2). Regarding OS, GG5 (HR = 1.69, *p* < 0.001), CRP (HR = 1.97, *p* < 0.001), LDH (HR = 2.80, *p* < 0.001), ALP (HR = 2.29, *p* < 0.001), and CHAARTED classification (HR = 1.92, *p* < 0.001) were determined as significant factors in univariate analysis. Multivariate analysis showed that GG5 (HR = 1.56, *p* < 0.001), CRP (HR = 1.52, *p* < 0.001), LDH (HR = 1.77, *p* < 0.001), ALP (HR = 1.62, *p* < 0.001), and CHAARTED classification (HR = 1.45, *p* = 0.008) were significantly associated with PFS (Table 3). These results suggested that GG5, CRP, LDH, and ALP were independent predictors for the prognosis of mHSPC.

**Table 2:** Univariate and multivariate analysis for PFS

	Univariate analysis			Multivariate analysis		
Parameter	HR	95% CI	p-value	HR	95% CI	p-value
Gleason grade						
Group 1–4	1 (Ref.)			1 (Ref.)		
Group 5	1.77	(1.45–2.15	< 0.001	1.69	(1.39–2.05)	< 0.001
CRP						
< 0.2	1 (Ref.)			1 (Ref.)		
>0.2	1.98	(1.66–2.38)	< 0.001	1.61	(1.33–1.94)	< 0.001
LDH						
< 300	1 (Ref.)			1 (Ref.)		
> 300	2.34	(1.83–2.95)	< 0.001	1.43	(1.10–1.85)	0.007
ALP						
< 500	1 (Ref.)			1 (Ref.)		
> 500	2.11	(1.75–2.54)	< 0.001	1.56	(1.26–1.92)	< 0.001
Visceral metastasis						
Absent	1 (Ref.)			1 (Ref.)		
Present	0.98	(0.77–1.24)	0.877	1.00	(0.78–1.29)	0.988
CHAARTED						
Low volume	1 (Ref.)			1 (Ref.)		
High volume	1.81	(1.50–2.19)	< 0.001	1.52	(1.22–1.89)	< 0.001
Regimen						
ADT/CAB	1 (Ref.)			1 (Ref.)		
Upfront ARSI	0.56	(0.40–0.78)	< 0.001	0.44	(0.31–0.61)	< 0.001

**Table 3:** Univariate and multivariate analysis for OS

	Univariate analysis			Multivariate analysis		
parameter	HR	95% CI	p-value	HR	95% CI	p-value
Gleason grade						
Group 1–4	1 (Ref.)			1 (Ref.)		
Group 5	1.69	(1.33–2.17)	< 0.001	1.56	(1.22―2.01)	< 0.001
CRP						
< 0.2	1 (Ref.)			1 (Ref.)		
>0.2	1.97	(1.57–2.49)	< 0.001	1.52	(1.19―1.93)	< 0.001
LDH						
< 300	1 (Ref.)			1 (Ref.)		
> 300	2.80	(2.12–3.65)	< 0.001	1.77	(1.30―2.37)	< 0.001
ALP						
< 500	1 (Ref.)			1 (Ref.)		
> 500	2.29	(1.81–2.87)	< 0.001	1.62	(1.24―2.10)	< 0.001
Visceral metastasis						
Absent	1 (Ref.)			1 (Ref.)		
Present	0.98	(0.77―1.24)	0.877	0.87	(0.62–1.20)	0.409
CHAARTED						
Low volume	1 (Ref.)			1 (Ref.)		
High volume	1.92	(1.52–2.44)	< 0.001	1.45	(1.10―1.90)	0.008
Regimen						
ADT/CAB	1 (Ref.)			1 (Ref.)		
Upfront ARSI	0.86	(0.55–1.35)	0.509	0.68	(0.43―1.07)	0.087

### Risk classification using four significant factors; GG5, CRP, LDH and ALP

**Fig. 1:**
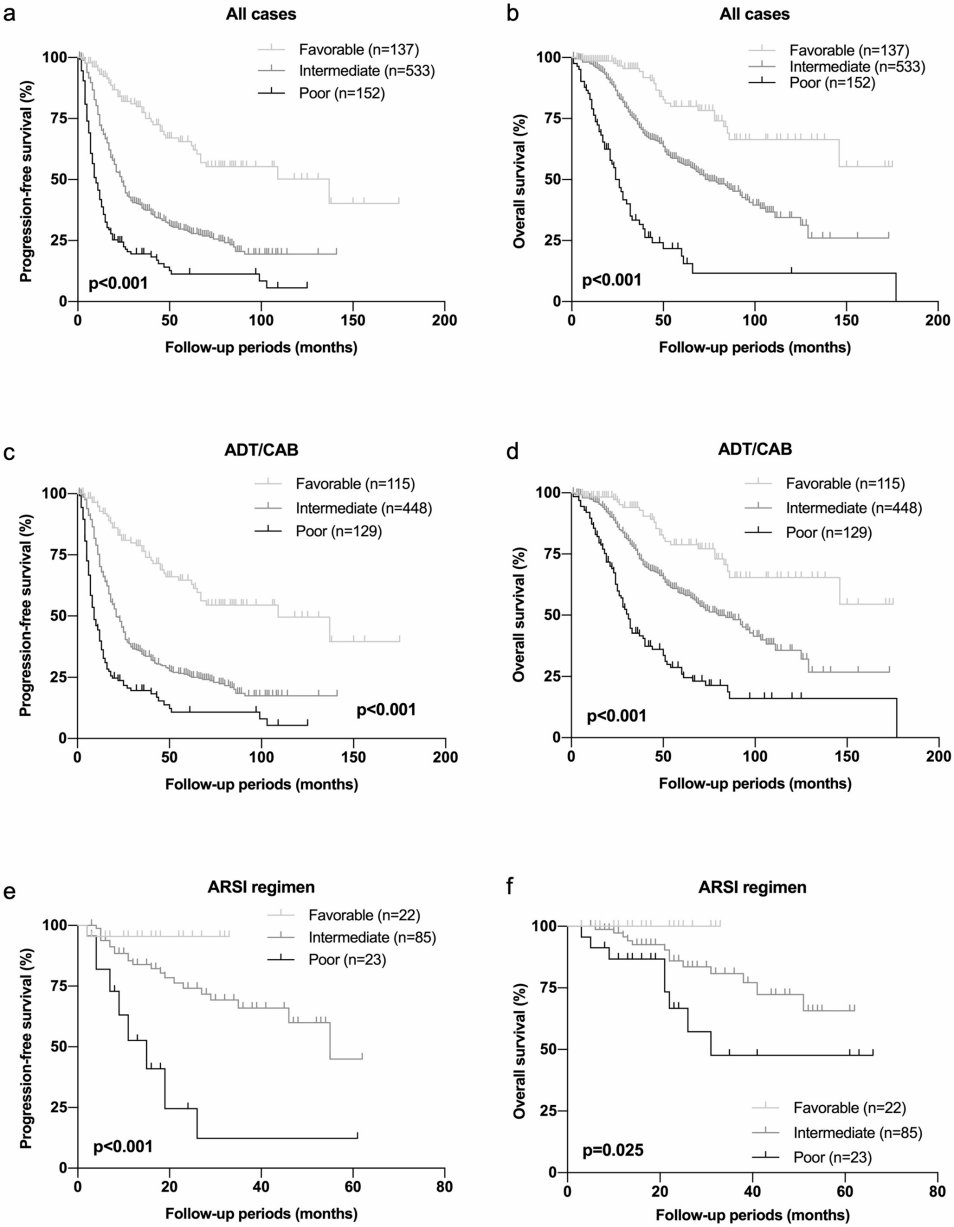
Progression free survival (PFS) and overall survival (OS) according to the risk classification using Gleason grade group, CRP, Alb, LDH, and ALP. p-value was estimated by log-rank test and *p* < 0.05 was determined as statistically significant

Among the predictors for OS in multivariate cox proportional hazards model, GG5, CRP, LDH, and ALP were determined as the most suitable factors by the stepwise regression. According to these four parameters, the patients were divided into three group including favorable (none of risk factors), intermediate (one or two risk factors) and poor (more than three risk factors). When the OS and PFS were investigated, the survival curves were significantly stratified according to the risk classification. The median PFSs were 137 months in favorable, 24 months in intermediate and 10 months in poor risk group, respectively (*p* < 0.001, Fig. 1a). The median OSs were not reached in favorable, 84 months in intermediate and 31 months in poor risk group, respectively (*p* < 0.001, Fig. 1b). When the risk classification was validated in patients who received ADT/CAB or upfront ARSI regimen respectively, the same tendency was observed. In patients who received ADT/CAB, the median PFSs were 109 months in favorable, 21 months in intermediate, and 9 months in poor risk group (*p* < 0.001, Fig. 1c) and the median OSs were not reached in favorable, 80 months in intermediate, and 31 months in poor risk group (Fig. 1d). In patients who received upfront ARSI regimen, the median PFSs were not reached in favorable, 55 months in intermediate, and 15 months in poor risk group (Fig. 1e) and the median OSs were not reached in favorable, not reached in intermediate, and 31 months in poor risk group (*p* = 0.025, Fig. 1f). These results suggested that GG5, CRP, LDH, and ALP were important risk factors in mHSPC regardless of treatment regimens.

### “Low-volume” disease is not “Low-risk” disease

**Fig. 2:**
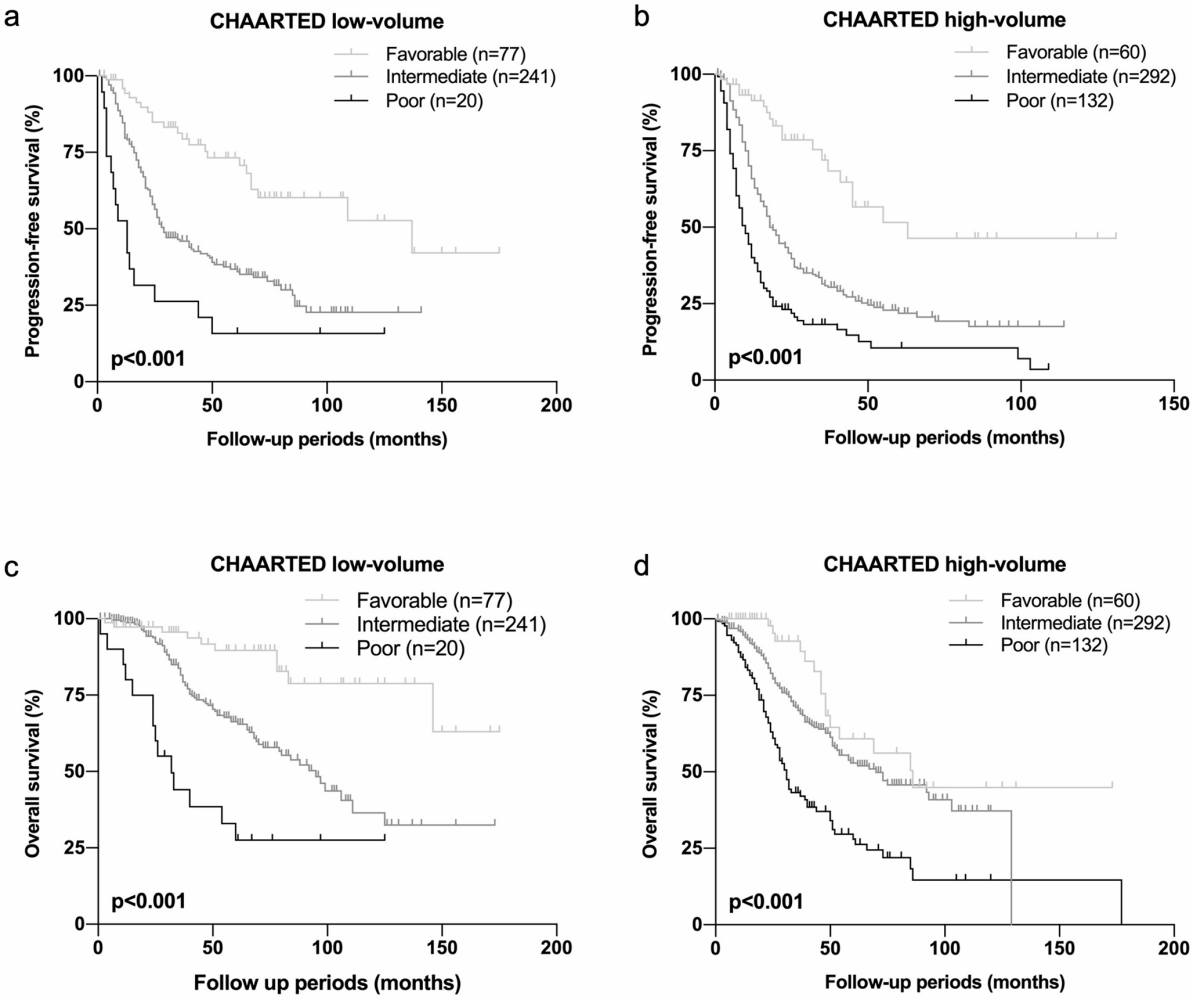
Progression free survival (PFS) and overall survival (OS) in patients with low- or high-volume disease in CHAARTED criteria, according to the risk classification using Gleason grade group, CRP, Alb, LDH, and ALP. p-value was estimated by log-rank test and *p* < 0.05 was determined as statistically significant

Furthermore, the risk classification was applied on the patients with low- or high-volume disease in CHAARTED criteria. Of interest, worse prognosis was found in poor risk group patients with low-volume disease and favorable prognosis was found in favorable risk group patients with high-volume disease. The median PFSs were 137 months in favorable, 29 months in intermediate and 17 months in poor risk group in low-volume disease (*p* < 0.001, Fig. 2a). In high-volume disease, the median PFSs were 63 months in favorable, 18 months in intermediate and 10 months in poor risk group (*p* < 0.001, Fig. 2b). Likewise, the median OSs were not reached in favorable, 95 months in intermediate and 32 months in poor risk group (*p* < 0.001, Fig. 2c) in low-volume disease. In high-volume disease, the median OSs were 86 months in favorable, 70 months in intermediate and 30 months in poor risk group (*p* < 0.001, Fig. 2d). These results suggested that the certain patients with high risk factors had aggressive potentials even though with low metastatic burdens.

### Differential behaviors between patients with different combination of risk factors

**Fig. 3:**
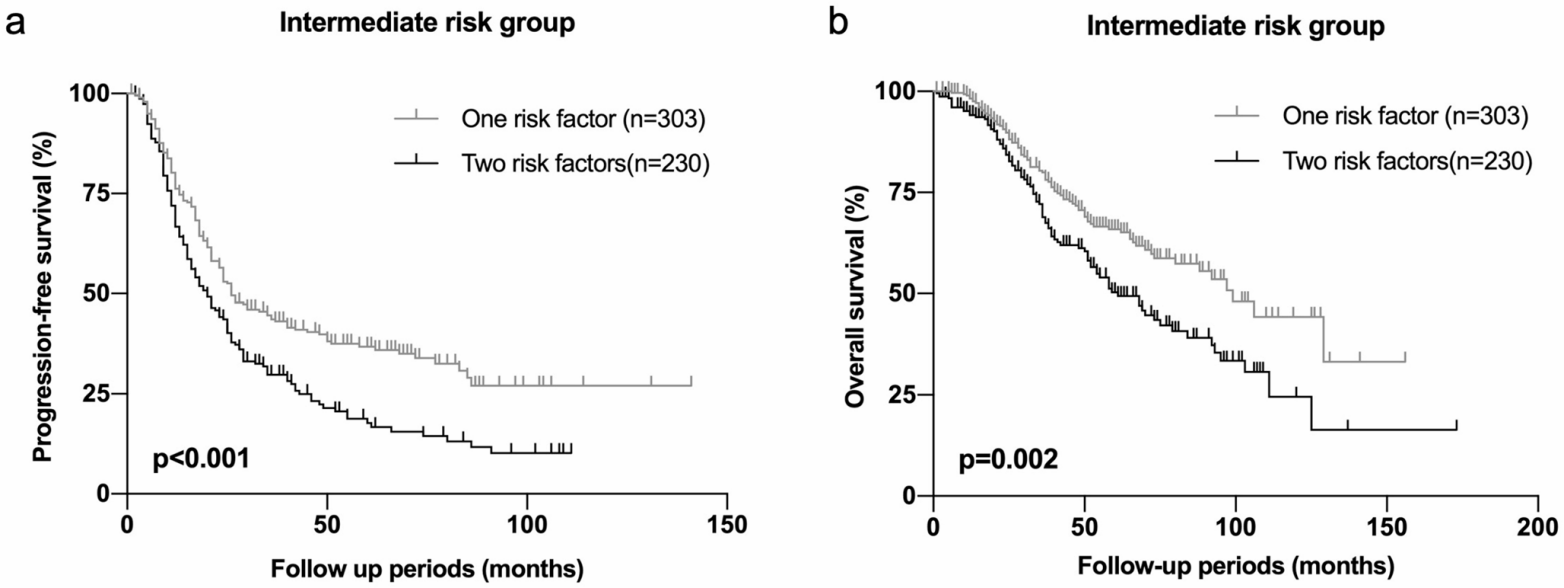
Progression free survival (PFS) and overall survival (OS) in patients with intermediate risk group, according to the number of risk factors including Gleason grade group, CRP, Alb, LDH, and ALP. *p*-value was estimated by log-rank test and *p*<0.05 was determined as statistically significant

In the risk classification using four significant parameters including GG5, CRP, LDH, and ALP, 533 (64.8%) out of 822 patients corresponded to intermediate risk group. For further detailed analyses, we performed whether different combination of risk factors in the intermediate risk group, that accounted the main bulk in this cohort. The patients with intermediate risk group had one or two of risk factors among GG5, LDH, ALP, and CRP. When focused on each parameter, only ALP was associated with PFS and OS. The other factors (GG5, LDH and CRP) were not associated with PFS nor OS (Supplementary Fig. S3). Next, PFS and OS were evaluated according to the number of risk factors. The patients with one risk factor had significantly better PFS (*p* < 0.001, Fig. 3a) and OS (*p* = 0.002, Fig. 3^3^) than the patients with two risk factors. These results suggested that the number of risk factors might be important when evaluating potential risk of mHSPC.

## Discussion

In this study, we evaluated various clinicopathological parameters to determine the significant predictors of patients with mHSPC. It has been reported that inflammatory and nutritional parameters were associated with the aggressiveness of PCa and that suggested the usefulness of certain index to predict the prognosis of mHSPC patients who underwent ADT [10, 16]. The survival analyses using individual factors revealed that T stage, N stage, Gleason score, EOD score and biochemical parameters including Alb, CRP, LDH and ALP were associated with PFS and OS. Also, similar results were obtained that high-volume disease in CHAARTED criteria had worse prognosis in our cohort. Previously, inflammatory, and nutritional indices that were calculated by blood counts or biochemical examinations were suggested as helpful indicators when evaluated the potential progressive risk of PCa [17–19]. The single parameter might be useful because of its simplicity. In the meantime, the risk classification should be determined from simple values among extensive candidates involving physical, biochemical, and histological factors. In our study, multivariate analyses demonstrated that the Gleason grade group 5, serum CRP, LDH, and ALP were determined as independent predictors for OS in patients with mHSPC. These four factors were obtained by prostate needle biopsy and blood biochemical examinations and should be evaluated in every case. In addition, the number of these risk factors were significantly correlated with the prognosis.

The number of bone metastasis was included in CHAARTED and LATITUDE criteria, considering that the correlation between metastatic burden and the tumor progression. The EOD score was one of representative parameters to assess the burden of bone metastases [14, 15]. It has been reported that EOD score was determined as the independent predictor and was used in the risk classification for patients with mHSPC [16, 20]. However, the EOD score was only for bone metastases, so that the other types of metastases including lymph nodes or visceral metastases were not taken accounted. The concept of tumor burden that was proposed to determine the differential prognostic factors in mHSPC patients was accounted in lymph node, bone, and visceral metastases [21, 22]. There was significant difference in the prognosis between low- and high- burden group, suggesting that the amount of metastatic tumors had important roles when evaluating the risk of mHSPC patients [23–25]. In our present study, EOD score was associated with higher CRP, LDH, and ALP levels but not determined as the independent factor in multivariate analysis, suggesting that the biochemical factors might complement the metastatic burden. CRP was the representative serum inflammatory marker, and LDH and ALP were also suggested as general surrogate tumor markers in PCa as well as the other types of malignant tumors. These biochemical markers were suitable for evaluating the risk of mHSPC because metastatic disease should be considered as a systemic disease. Besides, histological findings were also important to determine the risk of cancers, and Gleason grade group in PCa was one of the most sophisticated systems to classify the histological findings of cancers [26, 27]. Therefore, the risk classification including Gleason grade and general biochemical parameters was eligible for evaluating the potential prognosis of treatment naïve mHSPC patients. When our risk classification was applied into patients with low-volume disease in CHAARTED criteria, the significant difference was found between favorable, intermediate, and poor risk group. Based on our study, biochemical parameters could be prior to the other factors when the risk levels of mHSPC was assessed.

There were limitations in this study. It was the retrospective study performed across multiple institutions, that might imply the treatment strategy was not uniform. This study includes patients that were not indicated upfront AR signal inhibitors (ARSIs) at the time of diagnosis. Our results should be interpreted on the present standard therapy with care because most of the patients underwent vintage hormone therapy using bicalutamide and ADT.

In conclusion, we suggested the risk classification using biochemical and histological parameters that were available during regular clinical managements. Gleason grade, CRP, LDH, and ALP have been well known as the significant parameters and were also helpful to predict clinical outcomes of mHSPC patients regardless of metastatic burden. Further large-scale population cased validations will be needed to obtain much convincing evidence.

## Supplementary Information

Below is the link to the electronic supplementary material.

Supplementary Material 1

## Data Availability

No datasets were generated or analysed during the current study.
