# Supplementary material for: Risk classification by pathological and biochemical prognostic factors determined by extensive exploration for metastatic hormone sensitive prostate cancer
Source: World J Urol. 2025 Aug 11;43(1):483. doi: 10.1007/s00345-025-05862-4 (PMC12339647; doi:10.1007/s00345-025-05862-4)

Supplementary information

Risk classification by pathological and biochemical prognostic factors determined by extensive exploration for metastatic hormone sensitive prostate cancer

Goto K, Kobatake K, Fukuoka K, Kagiyama Y, Hatayama T, Kirishima F, Yukihiro K, Kurimura Y, Ikai T, Saito K, Shirane S, Yasumoto H, and Hinata N

Figure S1

Progression free survival (PFS) that were evaluated by Kaplan-Meier method according to clinical parameters. p-value was estimated by log-rank test and $p<0.05$ was determined as statistically significant. BMI; body mass index, PSA; prostate specific antigen, EOD; extent of disease, Hb; hemoglobin, Alb; albumin, CRP; C-reactive protein, AST; aspartate transaminase, ALT; alanine transaminase, LDH; lactate dehydrogenase, ALP; alkaline phosphatase.

Figure S2

Overall survival (OS) that were evaluated by Kaplan-Meier method according to clinical parameters. p-value was estimated by log-rank test and $p<0.05$ was determined as statistically significant. BMI; body mass index, PSA; prostate specific antigen, EOD; extent of disease, Hb; hemoglobin, Alb; albumin, CRP; C-reactive protein, AST; aspartate transaminase, ALT; alanine transaminase, LDH; lactate dehydrogenase, ALP; alkaline phosphatase.

Figure S3

Progression free survival (PFS) and overall survival (OS) in intermediate risk group according to clinical parameters. p-value was estimated by log-rank test and $p<0.05$ was determined as statistically significant. LDH; lactate dehydrogenase, ALP; alkaline phosphatase, CRP; C-reactive protein.

Supplementary Figure S1

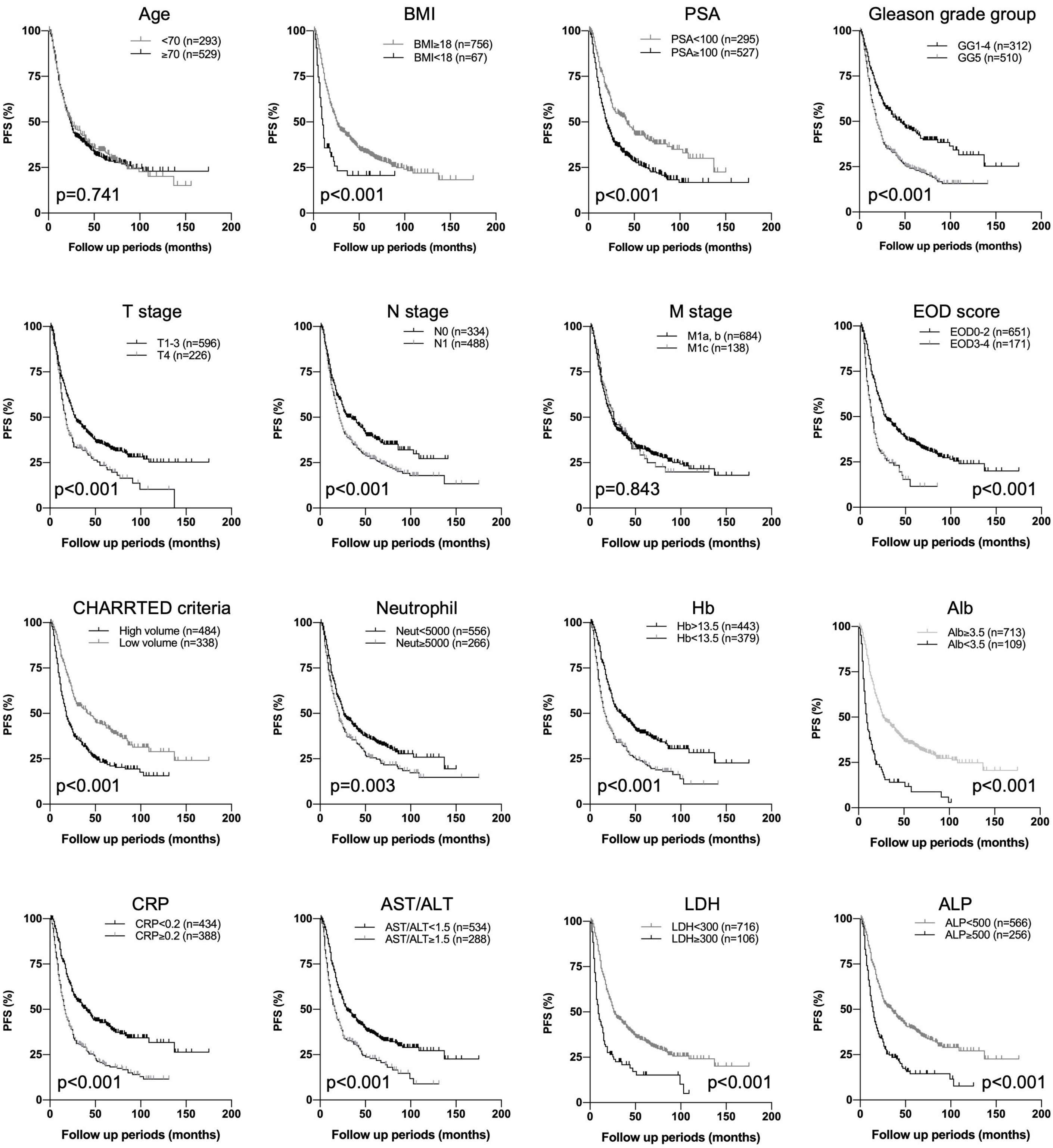

Supplementary Figure S2

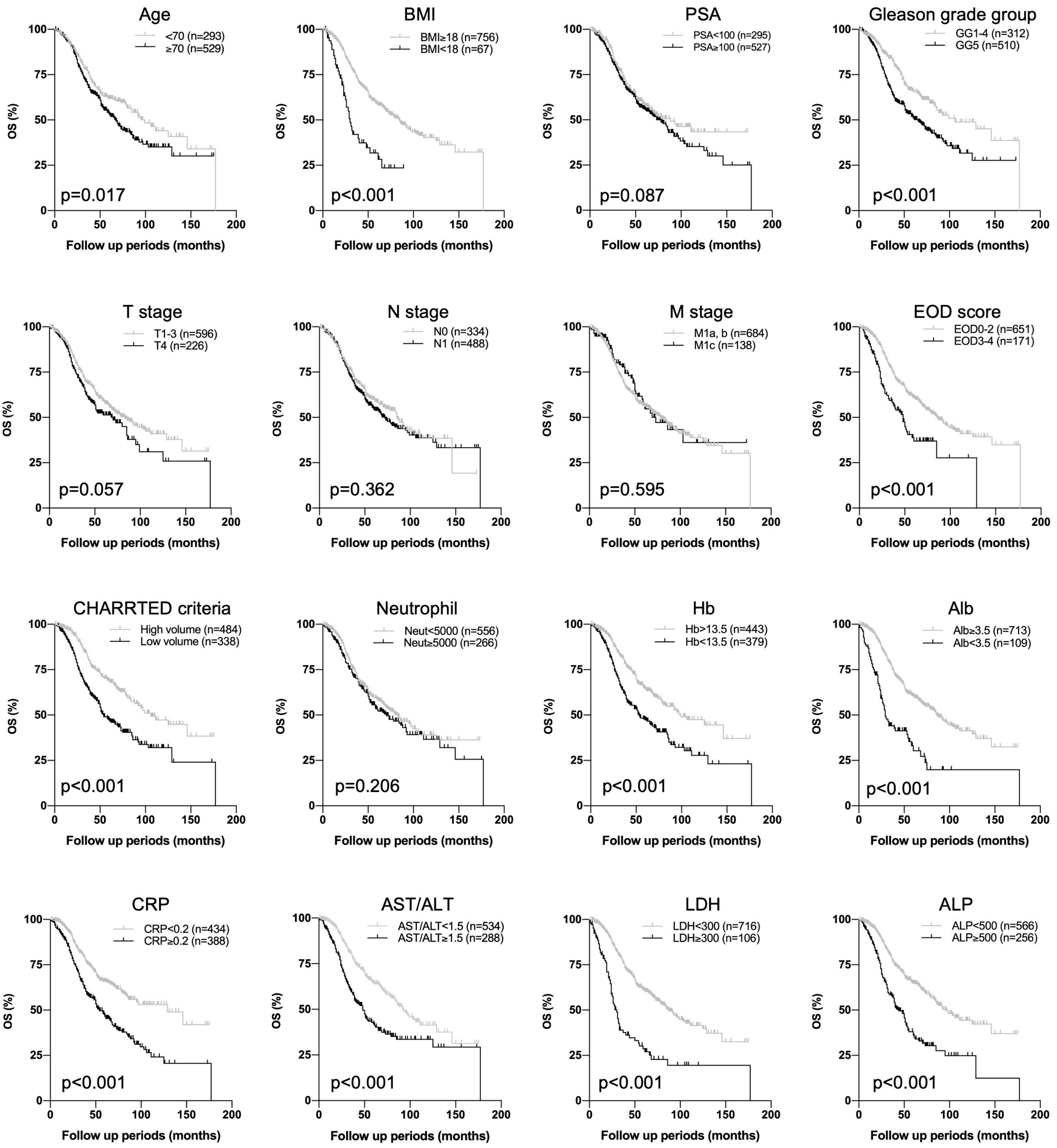

Supplementary Figure S3

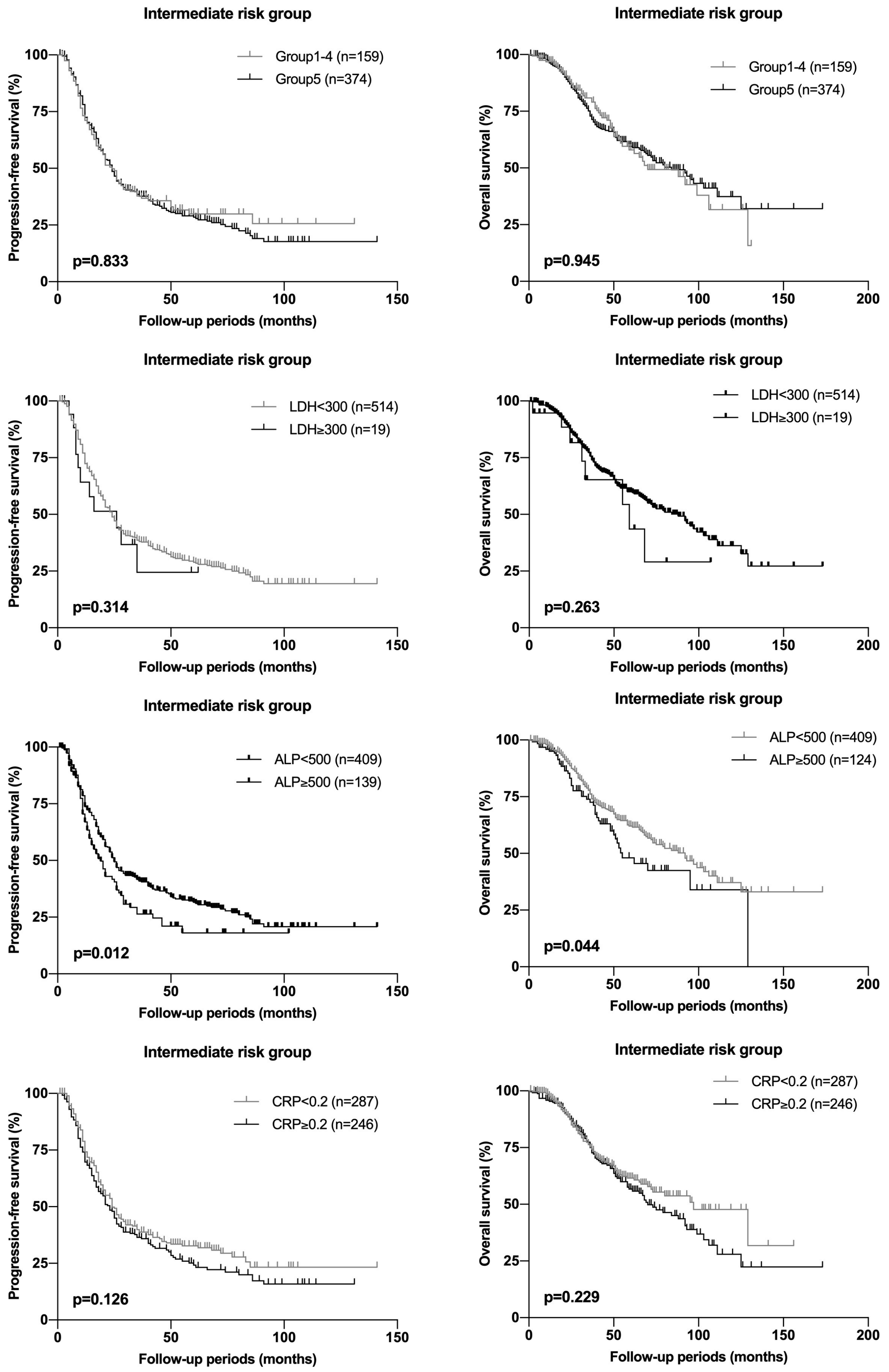

Supplement: Supplementary file 1 — Supplementary Material 1 [file 345_2025_5862_MOESM1_ESM.pdf]
